# Mechanistic target of rapamycin (mTOR): a point of convergence in the action of insulin/IGF-1 and G protein-coupled receptor agonists in pancreatic cancer cells

**DOI:** 10.3389/fphys.2014.00357

**Published:** 2014-09-23

**Authors:** Enrique Rozengurt

**Affiliations:** Division of Digestive Diseases, Department of Medicine, CURE: Digestive Diseases Research Center, David Geffen School of Medicine, Molecular Biology Institute, University of California at Los AngelesLos Angeles, CA, USA

**Keywords:** Akt, PI3K, PKC, S6 kinase, neurotensin

## Abstract

Pancreatic ductal adenocarcinoma (PDAC), the most common form of pancreatic cancer, is one of the most lethal human diseases. PDAC is now the fourth leading cause of cancer mortality in both men and women and deaths due to PDAC are projected to increase dramatically. Novel targets and agents for chemoprevention are urgently needed and will most likely arise from a more detailed understanding of the signaling mechanisms that stimulate the promotion and progression of sub-malignant cells into pancreatic cancer cells and from the identification of modifiable risk factors for PDAC. Many epidemiological studies have linked obesity and long-standing type 2 diabetes mellitus (T2DM) with increased risk and worse clinical outcomes for developing PDAC. These diet-related metabolic disorders are multifaceted but characterized by peripheral insulin resistance, compensatory overproduction of insulin and increased bioavailability of insulin-like growth factor-1 (IGF-1). Mounting evidence indicates that the insulin/IGF-1 receptor system plays a critical role in PDAC development and multiple studies support the notion that crosstalk between the insulin receptor and heptahelical G protein-coupled receptor (GPCR) signaling systems is an important element in the biological responses elicited by these signaling systems, including cell proliferation. This article highlights the central role of the mechanistic target of rapamycin (mTOR) in mediating crosstalk between insulin/IGF-1 and GPCR signaling in pancreatic cancer cells and proposes strategies, including the use of metformin, to target this signaling system in PDAC cells.

Pancreatic ductal adenocarcinoma (PDAC), the most common form of pancreatic cancer, is one of the most lethal human diseases. Indeed, the overall 5-year survival rate is a dismal 6% and the median survival period of 4–6 months. The incidence of this disease in the US is estimated to increase to more than 44,000 new cases in 2014 and is now the fourth leading cause of cancer mortality in both men and women (Siegel et al., 2014). Total deaths due to PDAC are projected to increase dramatically (Rahib et al., 2014). Novel targets and agents for chemoprevention are urgently needed and will most likely arise from a more detailed understanding of the signaling mechanisms that stimulate the promotion and progression of sub-malignant cells into pancreatic cancer cells and from the identification of modifiable risk factors for PDAC. In this context, it is recognized that PDAC arises from the progression of precursor lesions, the most common of which are pancreatic intraepithelial neoplasias (PanINs). Progression from these non-invasive lesions to invasive cancer is associated with the accumulation of genetic alterations (Murphy et al., 2013), including activating mutations in the *KRAS* oncogene which appears in ~90% of PDACs as well as inactivating mutations in tumor suppressors genes, including p53, p16, and SMAD4 (Murphy et al., 2013). It is generally accepted that progression of pancreatic carcinogenesis requires dysregulation of a set of signaling pathways leading to sustained cell proliferation (Jones et al., 2008). The focus of this brief article is on the central role of the mechanistic/mammalian target of rapamycin (mTOR) in mediating insulin/IGF-1 and G protein-coupled receptor (GPCR) signaling leading to proliferation of pancreatic cancer cells. Subsequently, strategies to target this pathway in PDAC cells are proposed.

## Obesity, type 2 diabetes, and PDAC

In addition to smoking, chronic pancreatitis and a family history of PDAC (Kolodecik et al., 2014), many epidemiological studies have linked obesity and long-standing type 2 diabetes mellitus (T2DM) with increased risk and worse clinical outcomes for developing PDAC (Arslan et al., 2010; Giovannucci et al., 2010). These diet-related metabolic disorders are multifaceted but characterized by peripheral insulin resistance, compensatory overproduction of insulin and increased bioavailability of IGF-1 (Alemán et al., 2014). Given the complex organization of the pancreatic microcirculation, locally overproduced insulin by β cells is thought to act directly on insulin receptors expressed by exocrine pancreatic cells. The highly related insulin-like growth factor-1 (IGF-1) receptor (IGF-1R) and hybrids of IGF-1R and insulin receptors can also be activated by insulin (Taniguchi et al., 2006), in particular at the high concentrations of intra-pancreatic insulin. Accordingly, PDAC cells express insulin and IGF-1 receptors and over-express insulin receptor substrate (IRS)-1 and IRS-2 and PDAC (but not normal) tissue expresses activated IGF-1R and IGF-1 (Rozengurt et al., 2010). Silencing the expression of IGF-1R in pancreatic cancer cells inhibits their growth and metastasis (Subramani et al., 2014) and the beneficial effects of calorie restriction in pancreatic cancer models appear mediated through the IGF-1/IGF-1R axis (Harvey et al., 2014). Reciprocally, the promoting effects of high calorie diet have been associated with an increase in the circulating levels of insulin and IGF-1 (Dawson et al., 2013). Interestingly, IGF-1 and orthotopically transplanted PDAC growth were decreased in liver-specific IGF-1-deficient mice and restored by IGF-1 administration (Lashinger et al., 2013). Inactivation of p53, as seen during the progression of 50–75% of PDAC, has been recognized to potently up-regulate the insulin/IGF-1 pathway (Feng and Levine, 2010) and gene variations in the IGF-1 signaling system have been associated with worse survival in PDAC (Dong et al., 2010). Collectively, these studies underscore the significance of the insulin/IGF-1 signaling pathway in PDAC development. Accordingly, elucidation of the signaling pathways triggered by insulin/IGF-1 and the crosstalk mechanisms between the insulin/IGF-1R and other signaling pathways in PDAC cells is likely to facilitate the identification of new targets for therapeutic and chemo-preventive interventions.

## Insulin/IGF-1 signaling, PI3K/Akt/mTOR and PDAC

**Figure 1:**
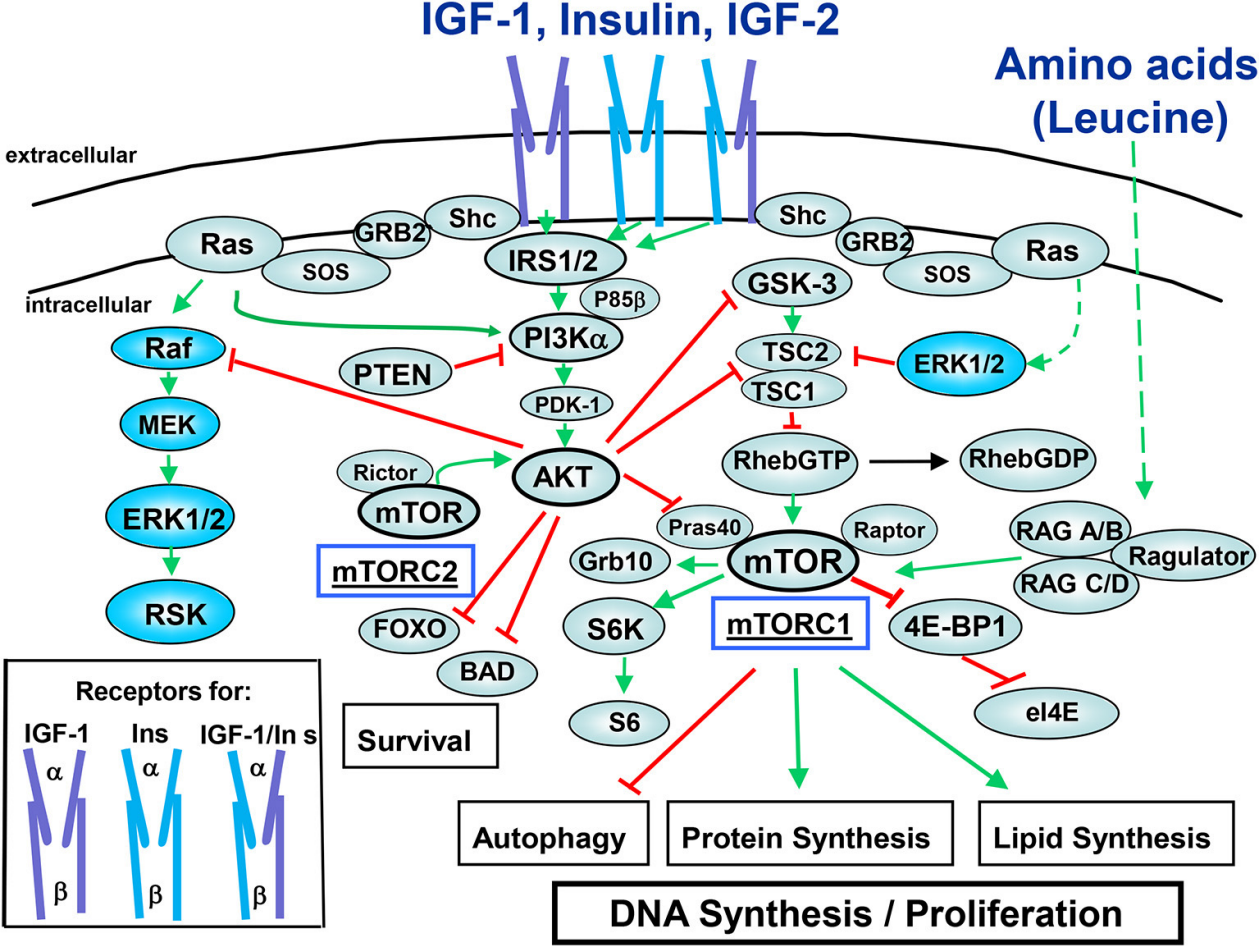
**Insulin/IGF-1 signaling pathways**. The receptors for the peptides of the insulin family peptides consist of ligand-binding α chain and tyrosine kinase-containing β chain (cartoons in the box). Insulin binds to the insulin receptors (InsR) with high affinity while it binds to IGF-1R at higher concentrations. Insulin also binds to hybrid receptors (IGF-1R/InsR). IGF-1 binds to the IGF-1R and to hybrid receptors with high affinity. IGF-2 binds to the InsR-A, IGF-1R, and IGF-1R/InsR-A hybrid receptor. For the sake clarity, negative feedback loops mediated by downstream components of the pathway (e.g., mTORC1, S6K) that restrain the activity of upstream components (e.g., IGF-1R, IRS) have not been included. The signaling network depicted in this figure is discussed in the text. Note that the IGF-1R and hybrid IGF-1R/InsRs couple more efficiently to Shc/Grb2/SOS providing an explanation for the increased ability of IGF-1 to induce ERK activation as compared with insulin. Green lines indicate stimulatory interactions while red lines indicate inhibitory interactions.

In most cells, binding of insulin to its tetrameric receptor induces activation of the receptor tyrosine kinase and autophosphorylation, followed by docking and tyrosine phosphorylation of adaptor proteins, including insulin receptor substrates (IRS 1–4) and Shc which propagate downstream signals (Metz and McGarry Houghton, 2011). The insulin receptor exhibits a high degree of homology with the IGF-1R, especially in their tyrosine kinase domains. Furthermore, the insulin and IGF-1 receptors form heterodimers that bind IGF-2, another ligand of the IGF family produced by cancer cells. As illustrated in Figure 1, a key insulin/IGF1R-induced pathway via IRS is class I phosphatidylinositol 3-kinase (PI3K)/Akt/mTOR (Taniguchi et al., 2006; Zoncu et al., 2011). PI3K catalyzes the synthesis of phosphatidylinositol (3,4,5)-trisphosphate (PIP_3_), a membrane lipid second messenger that coordinates the localization and activation of downstream effectors, including the isoforms of the Akt family (Franke, 2008). The Akts possess a PH domain and conserved residues (Thr^308^ and Ser^473^ in Akt1, the most commonly expressed isoform in normal cells) which are critical for Akt activation. Specifically, Akt translocated to the plasma membrane in response to products of PI3K, is activated by phosphorylation at Thr^308^ in the kinase activation loop and at Ser^473^ in the hydrophobic motif. The PI3K/Akt/mTOR pathway plays a pivotal role in promoting the proliferation and survival of PDAC cells (Asano et al., 2005), is activated in pancreatic cancer tissues, and limits catabolic processes, including autophagy (Lee et al., 2010). Interestingly, the Akt2 gene is amplified or activated in a subset of pancreatic carcinomas (Ruggeri et al., 1998). Collectively, these findings imply that mTOR signaling plays an important role in obesity-induced pancreatic cancer and is a potential target for chemoprevention.

mTOR, a master regulator of cell metabolism, growth and proliferation, functions as a catalytic subunit in two distinct multi-protein complexes, mTORC1 and mTORC2 (Beauchamp and Platanias, 2013). mTORC1, characterized by the substrate binding subunit Raptor senses both nutrients and growth factors (Dibble and Manning, 2013). As indicated in Figure 1, mTORC1 phosphorylates and controls at least two regulators of protein synthesis, the 40S ribosomal protein subunit S6 kinase (S6K) and the inhibitor of protein synthesis 4E-binding protein 1 (4EBP1) which promote protein synthesis and plays a critical role in the regulation of cellular metabolism (Dibble and Manning, 2013). mTORC1 is acutely inhibited by rapamycin whereas mTORC2, which is characterized by Rictor and mSin1, is not inhibited by short-term treatment with this agent.

The heterodimer of the tumor suppressor tuberous sclerosis complex 2 (TSC2; tuberin) and TSC1 (hamartin) represses mTORC1 signaling by acting as the GTPase-activator protein for the small G protein Rheb (Ras homolog enriched in brain), a potent activator of mTORC1 in its GTP-bound state. Phosphorylation of TSC2 by Akt and/or ERK/p90RSK (at different sites) uncouples TSC1/TSC2 from Rheb, leading to Rheb-GTP accumulation and mTORC1 activation (Figure 1). The Rag GTPases (RAGA/B and RAGC/D), in conjunction with the adaptor Ragulator, activate mTORC1 in response to amino acids, by promoting mTORC1 translocation to lysosomal membranes that contain Rheb-GTP (Bar-Peled and Sabatini, 2014). Phosphatase and tensin homolog (PTEN) opposes PI3K by degrading PIP_3_ to PIP_2_ thereby inactivating Akt and mTOR signaling (Song et al., 2012). The adaptor protein Shc binds to autophosphorylated IGF-1R to stimulate Grb2/SOS-mediated Ras activation (GTP loading) leading to Raf/MEK/ERK activation (Figure 1). As will be discussed below, insulin/IGF-1-induced signaling cross-talks with pathways triggered through other receptors systems expressed by PDAC cells thereby forming complex networks.

In addition to be phosphorylated at multiple Tyr residues that promote downstream signaling, the IRS family is also phosphorylated at multiple serine and threonine residues that attenuate signaling and promote degradation. In this context, it is important that activation of the mTORC1/S6K axis inhibits IRS-1 function following its phosphorylation at multiple residues, including Ser^636/639^ by mTORC1 and Ser^307/636/1001^ by S6K (Tanti and Jager, 2009). Accordingly, treatment of PDAC cells with rapamycin caused a striking increase in Akt phosphorylation at Ser^473^ while exposure to active-site inhibitors of mTOR (e.g., KU63794 and PP242) abrogated Akt phosphorylation at this site in PDAC cells (Soares et al., 2013). Conversely, active-site inhibitors of mTOR caused a marked increase in ERK activation whereas rapamycin did not have any stimulatory effect on ERK activation in PDAC cells (Soares et al., 2013). These results imply that first and second generation of mTOR inhibitors promote over-activation of different pro-oncogenic pathways in PDAC cells, suggesting that suppression of feed-back loops should be a major consideration in the use of these inhibitors for PDAC therapy.

## Crosstalk between insulin/IGF-1 receptor and G protein-coupled receptor signaling systems in PDAC

Many studies support the notion that crosstalk between the insulin receptor and heptahelical GPCR signaling systems is implicated in a variety of normal and abnormal processes, including cardiovascular and renal pathologies in obesity, metabolic syndrome and T2DM. Many GPCRs and their cognate agonists also mediate autocrine/paracrine growth stimulation in a variety of cancer cells and dramatically synergize with insulin/IGF-1 in inducing mitogenic signaling (Rozengurt, 1986). A recent characterization of cancer genomes demonstrated frequent mutations in GPCRs and G proteins (Kan et al., 2010). Consequently, we hypothesized that crosstalk between insulin/IGF-1 receptor and GPCR signaling systems is also a mechanism for enhancing the development of pancreatic cancer (Rozengurt et al., 2010). Accordingly, PDAC cells and tissues express multiple mitogenic GPCRs, including receptors that recognize neurotensin, angiotensin II and substance P (Rozengurt et al., 2010) and a broad-spectrum GPCR antagonist inhibited the growth of PDAC cells *in vivo* (Guha et al., 2005). Using PDAC cells in culture, we demonstrated positive crosstalk between insulin receptor and GPCR signaling systems (Kisfalvi et al., 2009).

**Figure 2:**
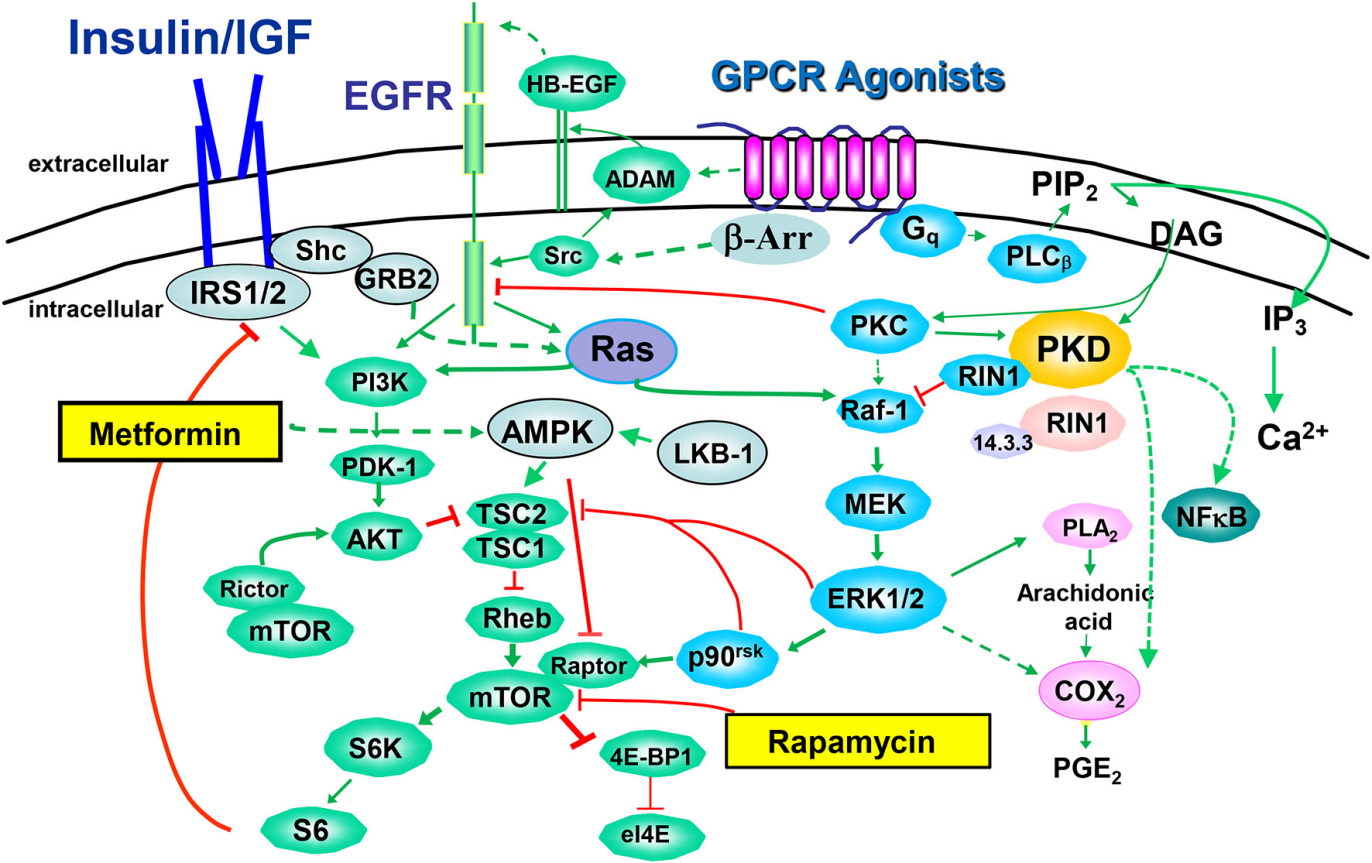
**Crosstalk between insulin/IGF-1 receptors and GPCR signaling systems**. The binding of an agonistic ligand to its cognate GPCR triggers the activation of multiple signal transduction pathways via heterotrimeric G proteins, including Gq/11. GPCRS also signal via arrestin (β-Arr) in a G protein-independent manner. A rapid increase in the activity of phospholipases C leads to the synthesis of lipid-derived second messengers, Ca^2+^ fluxes and subsequent activation of protein phosphorylation cascades, including PKC/PKD, Raf/MEK/ERK and Akt/mTOR/p70S6K. The EGFR has emerged as a transducer in the signaling by GPCRs, a process termed EGFR transactivation, and promoted by the release of heparin-binding epidermal growth factor (HB-EGF) through the activation of a disintegrin and metalloprotease (ADAM). The pathways stimulated by GPCRs are extensively interconnected by synergistic and antagonistic cross-talks that play a critical role in signal transmission, integration and dissemination. In this context, mTOR emerges as a critical point of convergence in the action of insulin/IGR-1R, EGFR, and GPCRs. Rapamycin, an allosteric inhibitor of mTORC1 and metformin, an inhibitor of mitochondrial function that indirectly (broken lines) stimulates AMPK, are also included.

Many GPCRs activate G proteins of the Gq family, promoting its dissociation into Gαq and Gβ γ and the exchange of GDP bound to Gαq for GTP (Rozengurt, 2007). The resulting GTP-Gαq complex activates the β isoforms of phospholipase C (PLC), identified as one of the “core” signaling pathways that undergo somatic alterations in nearly all pancreatic cancers (Jones et al., 2008). As shown in Figure 2, PLCβ produces second messengers that activate members of the protein kinase C (PKC) family which, in turn, phosphorylate and activate the protein kinases of the protein kinase D (PKD) family, including PKD1, PKD2, and PKD3 (Rozengurt et al., 2005). The PKC/PKD axis induces MEK/ERK/p90RSK activation, at least in part by direct phosphorylation of RIN1 and thereby potentiates K-Ras signaling (Rozengurt et al., 2005). In addition, PKDs can promote COX-2-mediated production of PGE2 which can bind to their own receptors after exiting the cells (Figure 2). PKDs are rapidly activated by GPCR agonists in PDAC cells (Guha et al., 2002; Rey et al., 2003a,b; Yuan and Rozengurt, 2008), are over-expressed in PDAC tissues (Harikumar et al., 2010) and PKD over-expression in PDAC cell lines promotes their proliferation (Kisfalvi et al., 2010) and invasion (Ochi et al., 2011). Furthermore, a novel PKD inhibitor blocks pancreatic cancer cell growth *in vitro* and *in vivo* (Harikumar et al., 2010).

GPCR agonists also stimulate mTORC1 through at least two converging mechanisms: EGFR transactivation and ERK-mediated phosphorylation of TSC2 (Rozengurt, 2007; Foster and Fingar, 2010; Rozengurt et al., 2010). Transactivation of the EGFR is mediated by the rapid generation of EGFR ligands through proteolysis of membrane-bound precursors proteins and via intracellular phosphorylation of EGFR mediated by Src (Santiskulvong and Rozengurt, 2007). The importance of EGFR has been demonstrated in transgenic mice models in which pancreas-specific deletion of EGFR prevented *Kras*-induced development of PDAC (Ardito et al., 2012).

We hypothesize that the concomitant activation of PI3K/Akt (through insulin/IGF-1 and EGF receptors), PKD/ERK (via agonist-induced Gq signaling) and mTORC1 (synergistically through PI3K/Akt induced by insulin/IGF-1R and EGFR and GPCR-stimulated ERK/p90RSK) in PDAC cells potently stimulates DNA synthesis and proliferation of these cancer cells, and thus provide potential targets for chemotherapeutic intervention (Figure 2). Since both the ERK and PI3K pathways are effectors of KRAS, activating mutations of *KRAS* reinforce the crosstalk between insulin/IGF-1 receptor and GPCR signaling systems, thereby increasing the robustness of the network induced by insulin/IGF-1 and GPCR agonists in pancreatic cancer cells.

## Metformin, AMPK, and PDAC

Metformin (1,1-dimethylbiguanide hydrochloride) is the most widely prescribed drug for treatment of T2DM worldwide. Although it has been in clinical use for decades, its precise molecular mechanism of action remains incompletely understood. The primary systemic effect of metformin is the lowering of blood glucose levels through reduced hepatic gluconeogenesis and improved insulin sensitivity by increasing glucose uptake in peripheral tissues, including skeletal muscles and adipose tissue (Shaw et al., 2005). Metformin also reduces the circulating levels of insulin and IGF-1 in both diabetic and non-diabetic patients (Berker et al., 2004; Goodwin et al., 2008).

At the cellular level, metformin indirectly stimulates AMP–activated protein kinase (AMPK) activation (Hawley et al., 2010), though other cellular mechanisms of action have been proposed, especially at high concentrations (Sahra et al., 2008; Kalender et al., 2010). Metformin does not act directly on AMPK but inhibits complex I activity of the mitochondrial respiratory chain (El-Mir et al., 2000; Owen et al., 2000), resulting in reduced ATP synthesis and increase in cellular AMP and ADP. AMPK is a conserved sensor of cellular energy being activated when ATP concentrations decrease and 5′-AMP concentrations increase (Kahn et al., 2005; Oakhill et al., 2011). Interestingly, AMPK is also implicated in the regulation of epithelial cell polarity (Mirouse et al., 2007), which is lost in advanced PanINs (Hingorani et al., 2003).

AMPK exists as a heterotrimer, composed of the catalytic kinase α subunit and two regulatory subunits, β and γ (Kahn et al., 2005). AMP directly binds to the AMPK γ subunit, causing allosteric activation and preventing dephosphorylation of Thr^172^ in the activation loop of the α subunit (Gowans et al., 2013). The tumor suppressor LKB-1/STK11 (Liver kinase B1/serine–threonine kinase 11) is the major kinase phosphorylating the AMPK activation loop. LKB-1/STK11 is mutated in the Peutz-Jegher syndrome (Kahn et al., 2005), characterized by predisposition to GI neoplasms, including PDAC.

AMPK is thought to inhibit mTORC1 function at three levels: (1) AMPK stimulates TSC2 function via phosphorylation on Ser^1345^ (Inoki et al., 2003, 2006; Shaw et al., 2004), leading to accumulation of Rheb-GDP (the inactive form) and thereby to inhibition of mTORC1 activation; (2) AMPK inhibits mTORC1 by direct phosphorylation of Raptor (on Ser^722^ and Ser^792^), which disrupts its association with mTOR (Gwinn et al., 2008); (3) Insulin/IGF-1-induced mTORC1 activation is also attenuated by AMPK by direct phosphorylation of IRS-1 on Ser^794^, a site that interferes with PI3K activation (Tzatsos and Tsichlis, 2007; Ning and Clemmons, 2010). Metformin, at high concentrations, also inhibits mTORC1 via AMPK-independent pathways, targeting Rag GTPases and/or REDD1 (Kalender et al., 2010; Ben Sahra et al., 2011). Since mTORC1 is a key site of signaling crosstalk in PDAC cells, we examined whether metformin opposes positive crosstalk between insulin/IGF-1 receptors and GPCR signaling systems in these cells.

In designing mechanistic experiments with metformin or other inhibitors of mitochondrial respiration such as the natural alkaloid berberine, it is important to use physiological concentrations of glucose in the culture medium. Cancer cells use aerobic glycolysis when the glucose concentration in the medium is very high but retain significant capacity of oxidative phosphorylation (Rossignol et al., 2004; Imamura et al., 2009; Vander Heiden et al., 2009). Thus, when cultured in regular DMEM (which contains 25 mM glucose), cells derive most of the ATP from glycolysis. In contrast, when the concentration of ambient glucose is physiological (~5 mM) and glucose uptake rates are lower, cells derive part of their ATP from mitochondrial oxidative phosphorylation (Vazquez et al., 2010) and hence, are more sensitive to mild inhibitors of mitochondrial function, like metformin. Our results demonstrated that metformin prevented mTORC1 signaling in PDAC cells (Kisfalvi et al., 2009) and that the inhibitory effect of low doses of metformin on mTORC1 was markedly enhanced when PDAC cells were cultured in medium containing physiological concentrations of glucose (Sinnett-Smith et al., 2013; Soares et al., 2013). In this context, most previous studies *in vitro* with multiple cell types have used high concentrations of this agent to elicit effects [e.g., 5–30 mM], a condition that can lead to off-target effects. In addition to inhibit mTORC1, our results demonstrated that metformin prevented ERK activation in PDAC cells (Soares et al., 2013). Interestingly, the effects of metformin on Akt and ERK activation are strikingly different from allosteric or active-site mTOR inhibitors in PDAC cells, though all these agents potently inhibited the mTORC1/S6K axis (Soares et al., 2013). Furthermore, administration of metformin inhibited the growth of aggressive PDAC cells in xenograft models (Kisfalvi et al., 2013). Collectively, these studies imply that metformin inhibits mitogenic signaling, including mTORC1, ERK, and proliferation in PDAC cells and raise the attractive possibility that this anti-diabetic agent could offer a novel approach for the chemoprevention of PDAC (Rozengurt et al., 2010; Yue et al., 2014).

In line with this possibility, a number of epidemiological studies suggested a link between administration of metformin and reduced incidence of a variety of cancers in T2DM patients, including PDAC (Li et al., 2009; DeCensi et al., 2010; Lee et al., 2011; Bodmer et al., 2012; Franciosi et al., 2013; Zhang et al., 2013). Interestingly, metformin use in T2DM patients with PDAC was associated to better survival (Sadeghi et al., 2012). However, a meta-analysis of nine observational studies showed a trend but failed to show a significant association between metformin and PDAC risk (Singh et al., 2013). Methodological limitations and biases that potentially exaggerate the beneficial effects of metformin in observational studies have been identified (Gandini et al., 2014). In any case, epidemiological associations do not establish causation, but support the need for understanding mechanism(s) of action and for prospective clinical studies. For example, it will be of great interest to test anti-cancer effects of metformin on PDAC cells with complex I mutations that render them hypersensitive to inhibitors (Birsoy et al., 2014).

The elucidation of the mechanism(s) by which metformin targets cancer cells is key for advancing the field as can lead to novel therapeutic strategies, including the identification of specific patient populations that ultimately will benefit from metformin administration, the generation of preliminary biomarker evidence of target inhibition, will stimulate the development of second generation drugs and the design of combinatorial interventions.

### Conflict of interest statement

The Associate Editor Guido Eibl declares that, despite having collaborated with the author Enrique Rozengurt, the review process was handled objectively and no conflict of interest exists. The author declares that the research was conducted in the absence of any commercial or financial relationships that could be construed as a potential conflict of interest.
